# Bridging the AI implementation gap in otolaryngology: A clinical commentary

**DOI:** 10.1177/20552076251396981

**Published:** 2025-11-18

**Authors:** James R. Burmeister, Ethan Dimock, Michael Haupert, Ismail Zazay

**Affiliations:** 1 Department of Foundational Medical Studies, Oakland University William Beaumont School of Medicine, Rochester, MI, USA; 2 Department of Otolaryngology, Corewell Health William Beaumont University Hospital, Royal Oak, MI, USA; 3 John Sealy School of Medicine, 12338 University of Texas Medical Branch, Galveston, TX, USA

**Keywords:** Artificial intelligence, otolaryngology, clinical integration, machine learning, workflow, explainability, bias, diagnostic tools

## Abstract

Artificial intelligence (AI) is moving rapidly from research into specialty clinical care. Otolaryngology (ENT), deeply reliant on imaging, endoscopy, and complex multimodal diagnostics, is positioned to benefit substantially, but faces unique barriers to real-world AI adoption. While prior commentaries have highlighted general obstacles such as data diversity, workflow integration, and explainability, this manuscript examines how these challenges manifest specifically in ENT subspecialties. Focusing on cochlear implant (CI) mapping, vestibular diagnostics, and voice/speech rehabilitation, we detail the distinctive workflow, regulatory, and medico-legal issues of AI in ENT. We provide a roadmap for closing the implementation gap, emphasizing the need for subspecialty-driven validation, tailored reporting standards, and collaborative governance. Ultimately, the responsible integration of AI in otolaryngology can serve as a model for translating advanced technologies into procedural, multidisciplinary fields.

## Introduction

Artificial intelligence (AI) is now a clinical imperative in medicine. In the past two years, the U.S. Food and Drug Administration (FDA) has cleared dozens of AI-enabled devices for diagnostic and workflow optimization across multiple specialties, including radiology and pathology.^1^ Yet otolaryngology (ENT), with its distinctive combination of imaging, endoscopy, audiometry, and speech/language evaluation, presents a set of implementation challenges that differ markedly from single-modality fields. General discussions of data bias, workflow integration, and explainability do not fully capture these specialty-specific issues.^2^ Here, we critically examine how the AI implementation gap in ENT is shaped by three high-impact clinical workflows: cochlear implant (CI) mapping, vestibular diagnostics, and voice/speech rehabilitation. We also address the unique regulatory and medico-legal landscape for AI deployment in ENT. The consequences of these challenges extend beyond technical barriers, failure to address specialty-specific gaps in AI integration can directly impact patient safety and worsen health inequities, especially for complex or underserved populations.

## Why ENT is different: specialty-specific AI barriers

Many AI models in medicine struggle with dataset diversity, but in ENT, the problem is magnified by multimodal and multidimensional data requirements.^3–5^ CI programming, for instance, demands integration of electrophysiological measures, imaging, audiometry, and patient-reported outcomes.^6,7^ Large, high-quality datasets linking these modalities are rare, and model generalizability remains poor. In vestibular diagnostics, a wide range of video-oculography devices, variable patient cooperation, and diverse protocols further limit the applicability of published models.^8–10^ In voice and speech rehabilitation, outcomes are influenced not only by anatomy and pathology but also by language, cultural factors, and comorbid neurologic or psychological conditions.^11^ Thus, AI systems trained on narrow, single-center data often fail to generalize to real-world, heterogeneous ENT populations.

In radiology, AI can often be layered onto image review workflows, but in ENT, clinical processes are interactive, multidisciplinary, and frequently procedure-based.^6,7^ For CI mapping, clinicians must interpret neural responses, speech perception scores, and real-time patient feedback, making “black box” AI recommendations both impractical and legally risky. Vestibular testing is not just about automated nystagmus detection but requires contextual diagnosis that incorporates patient history, medication effects, and bedside maneuvers.^8–10^ In voice clinics, therapy response tracking involves repeated acoustic, perceptual, and functional assessments, often requiring input from both surgeons and speech-language pathologists.^11^ AI systems that are not deeply embedded in these workflows risk being ignored or, worse, introducing clinical error.

While AI explainability is a universal challenge, in ENT the stakes are raised by the potential for irreversible harm, such as a missed glottic lesion, inappropriate CI programming, or misclassified vestibular emergencies.^12,13^ Unlike radiology, where AI errors may be mitigated by repeated imaging or further testing, a single missed airway lesion can have life-threatening consequences. Thus, explainable, auditable, and clinician-supervised AI is non-negotiable in ENT. Additionally, validation of AI outputs for endoscopy and voice analysis is complicated, as ground-truth labels often depend on expert consensus rather than strictly objective standards.

## Subspecialty deep dives: where the gaps are widest

CI mapping is a dynamic process that integrates electrophysiology, behavioral audiometry, and subjective experience.^6,7^ Although AI has shown promise in predicting outcomes and suggesting programming parameters, real-world CI patients often have comorbidities, anatomic variations, and psychological factors not represented in training datasets.^14,15^ Regulatory and liability issues also remain unclear: if an AI mapping recommendation results in a negative outcome, responsibility may be debated between the audiologist, surgeon, and software vendor.^14^ No clear regulatory pathway exists for “AI-assisted” neuroprosthetic mapping, and informed consent rarely addresses the risks or benefits of AI-driven programming.

AI-based nystagmus detection tools are promising, especially for rural and telehealth applications, but face substantial barriers.^8–10^ Device heterogeneity, variable video quality, and inconsistent eye-movement labeling complicate model validation and generalizability. Diagnoses such as central versus peripheral vertigo require contextual clinical integration beyond just eye-movement patterns. False negatives in AI-driven triage could delay neuroimaging for cerebellar stroke, raising both clinical and medico-legal risks.^16^ Furthermore, regulatory frameworks do not yet address continuous learning or model drift in these “diagnosis-assist” platforms, leaving significant uncertainty about post-market surveillance and accountability.

AI in voice and speech clinics aims to automate assessment, track progress, and even suggest therapy, but outcome measures are complex and multidimensional, including acoustic analysis, patient-reported quality, and speech-language pathology input.^11^ Cultural and linguistic factors, along with privacy and consent issues, present unique challenges. Importantly, voice data is highly identifying and can potentially be traced back to individuals, raising significant privacy concerns.^17^ Errors or biases in AI analysis could result in misdiagnosis or inappropriate therapy, particularly in underrepresented populations. ENT thus requires its own standards for explainability, reporting, and patient disclosure.

## Unique regulatory and medico-legal issues in ENT AI

AI in ENT is increasingly used for in-the-loop clinical interventions, such as real-time endoscopic decision support, intraoperative navigation, and neuroprosthetic adjustment.^18^ This introduces unique risks. Operator-dependent performance is a critical issue: AI for endoscopy or laryngoscopy is highly dependent on clinician technique, and variation in image acquisition can degrade model accuracy, potentially leading to missed lesions or false reassurance.^19^ If an AI system misses a critical airway lesion during endoscopy, legal liability may fall to the clinician, the institution, or the vendor, but current legal doctrine remains unsettled and likely varies by jurisdiction.^20^

Informed consent and documentation protocols for AI use are largely undeveloped in ENT, though regulatory guidance increasingly calls for transparency when AI is involved in diagnostic or therapeutic processes.^10,21,22^ Many AI tools used in ENT qualify as Software as a Medical Device (SaMD) and may change via continuous learning or software updates.^23^ This requires transparent version control, post-market monitoring, and explicit risk classification, all of which are currently underdeveloped in ENT practice.

## Roadmap for ENT-specific, responsible AI adoption

To close the implementation gap and foster safe, equitable AI adoption, ENT must move beyond generic guidelines. Large, multicenter, and multimodal datasets must be created for CI, vestibular, and voice applications, and external validation should be required before clinical use.^6–11^ Tools should be developed in close partnership with clinicians and embedded natively in electronic health records (EHRs), device consoles, and therapy software, rather than as standalone applications. ENT also needs its own standards for AI reporting and performance metrics, such as mapping-session duration, missed lesion rates, and validated improvement in voice handicap, building on frameworks like TRIPOD-AI and CONSORT-AI.^24,25^ Institutions should develop AI-specific consent forms, documentation protocols, and internal review boards for quality and safety, modeled after pharmacy and therapeutics committees. Education and accountability are also vital, AI competency milestones should be integrated into residency and continuing medical education, and quarterly audits for calibration drift, performance parity, and error tracking are essential for long-term safety and trust.

## How will AI change ENT day-to-day?

In community settings, AI-enabled smartphone otoscopy and automated sinus CT scoring may support generalists in triaging urgent cases.^3,26^ In rural clinics, voice-analysis apps may expedite identification of high-risk hoarseness, leading to faster referral and treatment.^27^ At tertiary centers, intraoperative AI overlays could highlight surgical landmarks or alert to at-risk structures during complex procedures.^6–11^ These advances will only benefit patients if tailored to the realities of ENT workflows and paired with clear oversight and accountability.

EHRs may soon flag hoarseness and generate risk scores before clinic visits, preload laryngoscopy templates, and suggest workup options. During surgery, endoscopic views may overlay real-time margin-probability heatmaps, and postoperatively, systems could draft notes, recommend Current Procedural Terminology (CPT) codes, and predict readmission risk, all integrated into clinical workflow.^28,29^ These tools must reinforce, not replace, clinician judgment and empathy. AI that complements rather than competes with physician expertise will define sustainable adoption. Over time, ENT specialists′ roles will shift toward oversight and curation, interpreting model outputs, troubleshooting inaccuracies, and collaborating on improvements. This stewardship echoes surgical safety culture, requiring transparency, monitoring, and shared accountability.

## Conclusion

The promise of AI in ENT cannot be realized without direct engagement with the field's specialty-specific challenges. ENT offers a template for translating advanced technology into procedural, multidisciplinary care. This is possible only&nbspif AI models are validated on real-world data, integrated into clinical workflows, and governed with ENT-specific legal and ethical safeguards. Moving beyond generic barriers, the field must critically assess and close the AI implementation gap within each ENT subspecialty, ensuring innovation delivers measurable benefits to patients and clinicians alike. Ultimately, failure to address these implementation gaps may not only delay progress but could also compromise patient safety and perpetuate disparities, particularly in high-stakes and underserved clinical scenarios. With continued cross-disciplinary collaboration, ENT can lead the way in safe, responsible AI adoption.
